# Flammability Characteristics of Thermally Modified Meranti Wood Treated with Natural and Synthetic Fire Retardants

**DOI:** 10.3390/polym13132160

**Published:** 2021-06-30

**Authors:** Milan Gaff, Hana Čekovská, Jiří Bouček, Danica Kačíková, Ivan Kubovský, Tereza Tribulová, Lingfeng Zhang, Salvio Marino, František Kačík

**Affiliations:** 1 Department of Wood Processing and Biomaterials, Faculty of Forestry and Wood Sciences, Czech University of Life Sciences Prague, Kamýcká 1176, Praha 6—Suchdol, 16521 Prague, Czech Republic; hanacekovska@gmail.com (H.Č.); jboucek@fld.czu.cz (J.B.); tereza.tribulova@gmail.com (T.T.); salviomarino@yahoo.it (S.M.); 2 Faculty of Wood Sciences and Technology, Technical University in Zvolen, T.G.Masaryka 24, 960 01 Zvolen, Slovakia; kacikova@tuzvo.sk (D.K.); kubovsky@tuzvo.sk (I.K.); kacik@tuzvo.sk (F.K.); 3 School of Civil Engineering, Southeast University, Nanjing 211189, China; lfzhang@yzu.edu.cn

**Keywords:** thermal modification, fire retardant, flammability characteristics, meranti

## Abstract

This paper deals with the effect of synthetic and natural flame retardants on flammability characteristics and chemical changes in thermally treated meranti wood (*Shorea* spp.). The basic chemical composition (extractives, lignin, holocellulose, cellulose, and hemicelluloses) was evaluated to clarify the relationships of temperature modifications (160 °C, 180 °C, and 210 °C) and incineration for 600 s. Weight loss, burning speed, the maximum burning rate, and the time to reach the maximum burning rate were evaluated. Relationships between flammable properties and chemical changes in thermally modified wood were evaluated with the Spearman correlation. The thermal modification did not confirm a positive contribution to the flammability and combustion properties of meranti wood. The effect of the synthetic retardant on all combustion properties was significantly higher compared to that of the natural retardant.

## 1. Introduction

Meranti (*Shorea* spp.) is one of the most widely used tropical hardwoods. It is relatively easy to process, and it has a course, fibrous structure with open pores. With a straight-grain consistency, meranti trees produce long, straight pieces of lumber. It is used for molding, structural elements, furniture, cabinets, window and door trim, and veneers for plywood. Meranti is one of the more affordable hardwoods, due in part to numerous subspecies, prolific growing characteristics, and availability. Similar to Teak (*Tectona grandis*), meranti and other hardwoods are resistant to damage from insects, fungus, and moisture decay. The wood is dimensionally stable and resistant to warping and twisting [1,2]. However, additional treatments are necessary to increase its fire resistance.

A very popular and ecological method is thermal modification of wood [3,4,5,6,7,8]. The purpose of thermal modification is to decrease the content of flammable substances present in the wood. Thermal treatments can also be a great alternative to improve physical and aesthetic properties, increase market value, and, consequently, the number of applications of wood from meranti [9,10]. Thermal treatment is normally performed at temperatures between 180 and 260 °C. Temperatures lower than 140 °C do not significantly affect the structure of the wood. Volatile and vapor substances are released under higher temperature. The process has to be very carefully optimized in order to not degrade the wood. Temperatures higher than 260 °C cause undesirable degradation [11,12].

According to the literature [13,14,15], the temperature is a key parameter of the process responsible for the highest effect of modification in the properties of thermally treated wood. Certified technologies that are used with commercial significance not only differ in applied temperature for a certain time, but also in ambient humidity, the use of a gaseous (air or nitrogen) or liquid (vegetable oils) environment, etc. All aforementioned factors, i.e., temperature, duration of thermal modification, and environment, have a certain impact on the results [16].

Surface treatment of wood with a flame-retardant finish was also applied in this research. With regard to environmental aspects of the thermal degradation of wood to retard the burning process, it should be noted that chemical treatment of these materials is approached on the basis of the “lesser risk” theory [17]. Most commercial fire-retardant formulations are not environmentally friendly [18,19,20].

For this reason, arabinogalactan was tested as a natural compound with a potential fire-retardant effect. It is well known that arabinogalactan is a biopolymer consisting of arabinose and galactose monosaccharides. In plants, it is a major component of many gums and it is often found attached to proteins. The resulting arabinogalactan protein functions as both an intercellular signaling molecule and a glue to seal plant wounds [21]. The big advantage is that it can be extracted from plant cells in its natural form using only water [22]. It is currently used in many ways, e.g., as an additive in food, animal feed, cosmetics, pharmacy, construction, pulp production, oil production, plant growth, etc. It generally plays an essential role in environmental preservation. With regard to this, and based on its properties [21,22,23], arabinogalactan is destined to become a suitable and effective flame retardant. This assessment was based on a comparison with the same previously thermally modified samples, treated with the synthetic fire-retardant system Flamgard Transparent. The main active ingredient of this commercial formulation is one of the most common fire-retardant inorganic chemicals—mono ammonium phosphate. Phosphates are some of the oldest known fire-retardant systems and they are usually included in most proprietary systems used for wood [24,25].

This article deals with the investigation of the thermal stability of thermally modified meranti wood that was subsequently treated with synthetic and natural fire retardants. The aim was to expand knowledge of thermal processes, disclose the possibilities of using arabinogalactan as an active ingredient in fire retardant formulations in gaining new facts on chemical reactions of thermal degradation, and to compare the efficiency of this environmentally friendly treatment with the application of a synthetic two-component formulation based on ammonium phosphates and an acrylic topcoat. The results of this study provide main flammability characteristics of modified meranti wood samples at temperatures of 160 °C, 180 °C, and 210 °C, namely, weight loss, burn rate, maximum burning rate, and ratio of the maxim um burning rate to time to reach the maximum burning rate.

## 2. Materials and Methods

### 2.1. Wood

We used meranti (*Shorea* spp.) wood for our research. Place of origin: Southeast Asia. Tree dimensions: height, 30 m; stem diameter, 1.5 m; dry wood density, 710 kg·m^−3^. Color: dark red-brown to red-brown. Wood hardness: 7.73 MPa. Samples were radially cut into 20 mm × 100 mm × 200 mm specimens. All wood samples were conditioned under specific conditions (relative humidity of 65% ± 3% and a temperature of 20 °C ± 2 °C) to achieve an equilibrium of moisture content of 12%. The conditioned samples were divided into three basic groups according to the fire-retardant application:

1. Thermal modification (TM);
2. Thermal modification + synthetic fire retardant (SFR);
3. Thermal modification + natural fire retardant (NFR).

### 2.2. Synthetic Fire Retardant

The flame retardant was Flamgard Transparent, which consists of two components. The first component is a flammable water-borne coating composition (a mixture composed of ammonium phosphates, foam-forming agent, oxalic and acetic acid, and fire-retardant additives), which is applied in three layers so that the total coat is at least 500 g·m^−2^. The second component is acrylic topcoat space 1818, which is only applied in one layer with a minimum coating of 80 g·m^−2^.

Both components were applied according to the manufacturer’s specifications. The curing of the individual layers took place at an ambient temperature of 20 °C. 

### 2.3. Natural Fire Retardant

Macromolecules containing arabinose and galactose have been found in most plant tissues. In some situations, they are isolated as polysaccharides free from associated protein; in other situations, they occur in covalent association with protein, either as proteoglycans, in which the protein component carries polysaccharide substituents [26,27], or as glycoproteins, in which the protein component is substituted by one or more oligosaccharide residues [28,29]. Arabinogalactans are a class of polysaccharides found in a wide range of plants; however, they are most abundant in plants of the genus Larix [30]. They have a highly branched structure. Their main chain consists of β-(1→3) linked galactose units. Approximately one-half of the side chains consist of β-(1→6)-linked dimers of galactopyranose; galactopyranose monomers account for about a quarter, and the remainder contains a major part of the polysaccharide’s arabinose in aggregates of two or more monomers [31,32,33]. Arabinose fragments mostly occur as side chains consisting of 3-O-substituted β-l-arabinofuranose residues and terminal residues of β-l-arabinopyranose, β-d-arabinofuranose, and α-l-arabinofuranose. However, arabinose fragments were also found in the main chain. Arabinogalactan has good solubility in cold water and the uniquely low viscosity of concentrated aqueous solutions [32,33].

### 2.4. Methods

#### 2.4.1. Thermal Modification

The thermal treatment was based on the Thermo Wood principle, developed by VTT (Espoo, Finland). The wood samples were treated in an S400/03 type thermal chamber (LAC Ltd., Rajhrad, Czech Republic). Three final temperatures of 160 °C, 180 °C, and 210 °C were set, and the treatment was carried out in a protective atmosphere (water steam) to prevent overheating and burning. The thermal modification was carried out in three basic stages (Table 1). All samples were dried in a ULM 400 oven (Memmert GmbH & amp; Co. KG, Schwabach, Germany) at T = 103 ± 2 °C to dry state. This condition facilitates the thermal modification of one group and also generates similar moisture for samples without thermal treatment, which is important from a comparative perspective.

The thermal modification of meranti wood was performed in the same way, according to the method described in publications on the wood species teak, padauk, and oak [34,35,36]. The density of the sample before and after the thermal modification is shown in Table 2.

#### 2.4.2. Physical Properties

For the sorting of sample quality, auxiliary criteria such as moisture content and density values can be used. These physical parameters should be sorted so that they do not affect the values of the basic assessment criteria. These two parameters were determined according to ISO 13061-2 [37] and ISO 13061-1 [38].

#### 2.4.3. Determination of Flammability Characteristics

The method of simulating actual fire conditions was performed according to the previously published procedure [38]. The heat source in this experiment was an open flame, patented, USBEC 1011/1 propane gas burner, supplied and regulated flame source (DIN-DVGW reg. NG-2211AN0133), 1.7 kW. Mass was measured by Mettler Toledo (MS1602S/MO1, Mettler Toledo, Geneva, Switzerland). Weight changes were recorded according to evaluation criteria performed with BalanceLink 4.2.0.1 (Mettler Toledo, Switzerland).

#### 2.4.4. Calculation of Given Characteristics for the Experiment

All flammability factors were evaluated in Statistica 13 (Statsoft Inc., Tulsa, OK, USA) using the two-way analysis of variance (ANOVA) and Spearman’s correlation. The sample density of meranti according to ISO 13061-2 (2014) [33] was determined together, and the moisture content of all samples according to ISO 13061-1 (2014) [39] was measured together before and after the thermal treatment.

#### 2.4.5. Chemical Analyses

All samples were mechanically decomposed into sawdust and a fraction size 0.5–1.0 mm was extracted according to ASTM D1107-96 [40]; the lignin content was determined according to Sluiter [41], holocellulose was determined using the method of [42], cellulose by the Seifert method [43], and the hemicelluloses content was calculated as the difference between holocellulose and cellulose. All measurements were performed on four replicates per specimen. The data were presented as percentages of the oven-dry weight of wood (odw) per unextracted wood.

The obtained values of the burning characteristics and percentage content of chemical wood components were evaluated in Statistica 13 (Statsoft Inc., Tulsa, OK, USA) with an analysis of variance (ANOVA) and Spearman’s correlation.

## 3. Results and Discussion

The average values of the chemical components of wood are shown in Table 3.

Table 4 shows the statistical significance of the effects of flammability properties on thermal modification and retardant treatment, including their interaction on meranti wood.

Based on the significance level of “*P*”, it is conclusive that both the flame retardant and the thermal modification or interaction of both factors have a strong statistical effect on the above characteristics.

The thermal modification in interaction with the retardant had a significant statistical effect on the burning characteristics (WL., BR., RMBR. and TRMBR.). However, for the rest of the characteristics (BR. and MBR.), thermal modification had a negligible effect.

In similar research, other authors also who dealt with this topic [40] showed that the degree of thermal treatment has no significant effect on any of the monitored characteristics in the case of spruce wood, i.e., WL.—600 s and 900 s (%), BR.—600 s and 900 s (%·s^−1^ × 10^−5^), RMBR. (%·s^−1^), (%) TRMBR. (s). These results show that none of the monitored characteristics are affected by thermal treatment of the wood and thermally modified wood is therefore suitable for use in timber structures just like wood that has not undergone thermal modification. The same team of authors in another study reported that thermal treatment only had a statistically significant effect on the values of monitored characteristics for weight loss—600 s (%) in the case of Teak wood.

Gašparík et al. [44] evaluated the effect of thermal modification and fire-retardant treatment on the flammability characteristics of oak wood. They concluded that thermal modification has an insignificant effect on the burn rate. Interaction of fire-retardant treatment and thermal modification has an insignificant effect on the MBR. Last but not least, fire-retardant treatment, thermal modification, and their interaction have an insignificant effect on the TRMBR.

**Figure 1:**
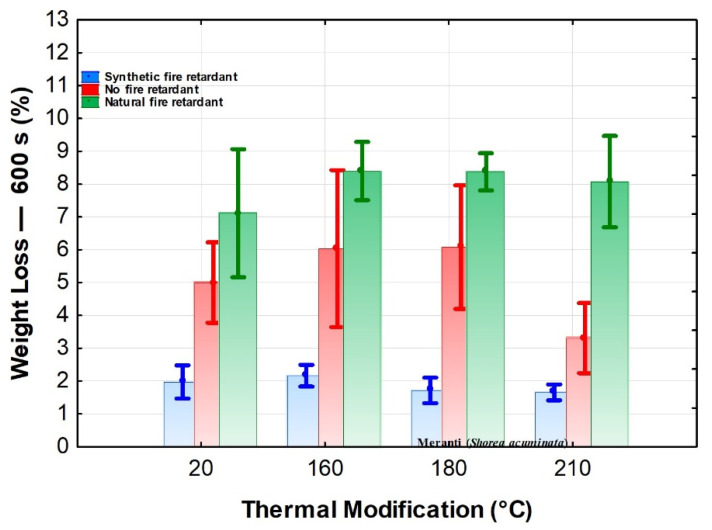
The effect of thermal modification and fire retardant on weight loss.

The significant effect of the flame retardant on weight loss is evident from the diagram shown in (Figure 1). The lowest average weight loss values were measured when a synthetic flame retardant was applied. The highest weight loss values were measured on samples treated with a natural flame retardant [44]. A similar observation in these trends was found for thermally modified oak wood and oak wood treated with a synthetic fire retardant [45]. The highest weight loss values for thermally treated wood were also found at 160 and 180 °C, and using a synthetic flame retardant at 160 °C. In the case of wood modified with a synthetic flame retardant, there were also minor differences in the decrease in weight loss at individual temperatures of the thermal modification.

**Figure 2:**
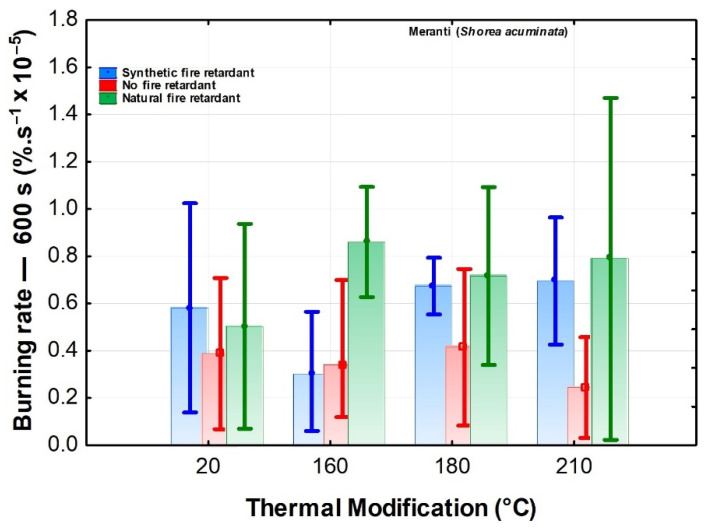
The effect of thermal modification and fire retardant on the burning rate.

In the case of burn rate characteristics—600 s (Figure 2), both the thermal modification and the flame retardant, as well as their interactions have an insignificant effect (however, the retardant is significant according to the table). In the above-mentioned research, the effect of thermal modification on the burn rate was not unambiguous [41]. Generally, the highest burn rate of thermally modified wood spruce, teak, and oak was observed at 180 °C, declining again at higher temperatures [44,45]. The expected effect of reduced burn rate with the application of flame retardants has not been confirmed in our case. It can be argued that, in almost all cases, the burn rate values of wood treated with flame retardants were equal to or higher than that of only thermally modified wood. Flame retardants reduce the flammability of materials by physical or chemical means: they may reduce or extinguish flame using an endothermic chemical reaction (occurs temperature limitation), or the pyrolysis modification process may reduce the amount of flammable volatile matter and increase production of fewer flammable substances that act as a barrier to protect others material. Retardants may act before ignition substances change during the pyrolysis process, they can also react in a flame and reduce the flammability of the substance during combustion, whether they can prevent the access of oxygen or heat to the hearth [46]. Synthetic and natural flame retardants exhibit similar behaviour, but only at 160 °C; the synthetic flame retardant provides better results, i.e., a lower burn rate.

**Figure 3:**
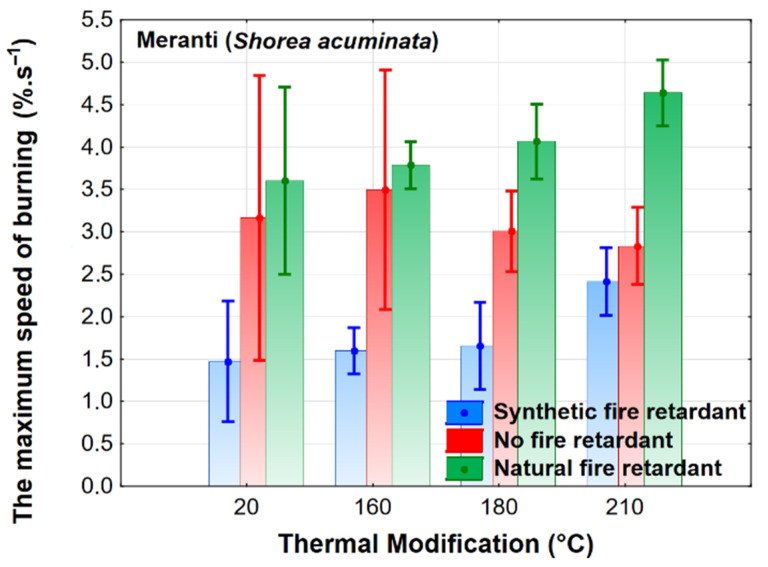
The effect of thermal modification and fire retardant on the maximum burn rate.

The burning rate is significantly affected by the application of a flame retardant (Figure 3). A more significant effect of the thermal modification can be seen at higher temperatures (180 and 210 °C) of thermal modification. The application of a synthetic flame retardant significantly reduces the values of this characteristic compared to only thermally modified wood, especially at temperatures of 20 °C, 160 °C, and 180 °C. The application of a natural flame retardant at lower temperatures has a very similar effect to that of thermally modified wood, deteriorating significantly at 180 and 210 °C, i.e., the maximum burn rate values increase. While, in this case, the effect of the thermal modification proved to be statistically insignificant (as in published works [44,45,47]), the effect of thermal modification of oak wood on the maximum burn rate was found to be statistically significant.

**Figure 4:**
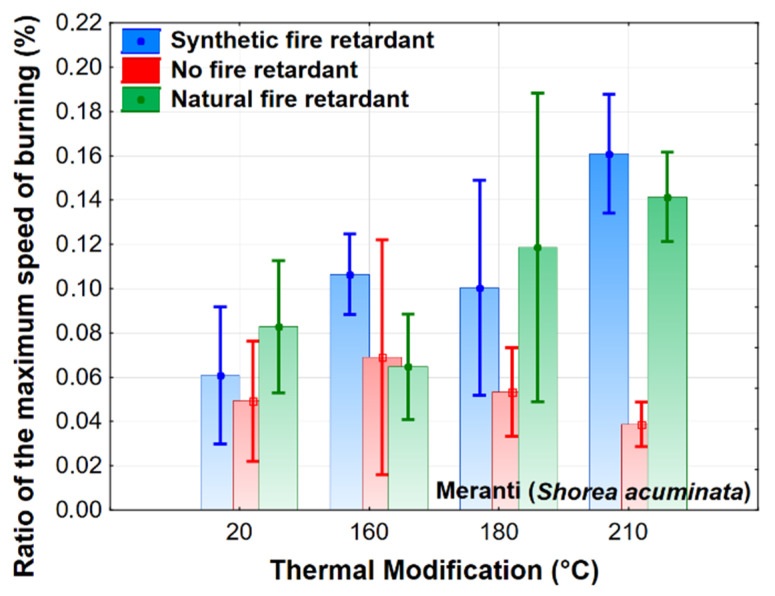
The effect of thermal modification and fire retardant on the ratio of the maximum burning rate.

The ratio of the maximum burn rate is significantly affected by the temperature of the thermal modification and the flame retardant and, therefore, also by the mutual interaction of these effects. The differences between thermally modified wood and wood treated with flame retardants only become apparent under higher thermal modification temperatures (Figure 4). From 180 °C, this characteristic has a downward trend in thermally modified wood, while, in wood treated with a flame retardant, it has a rising trend. The differences in the use of different types of flame retardants are insignificant in this case, the greatest relative differences are observed at a temperature of 160 °C, when the natural flame retardant causes a lower ratio of the maximum burn rate of the examined wood.

**Figure 5:**
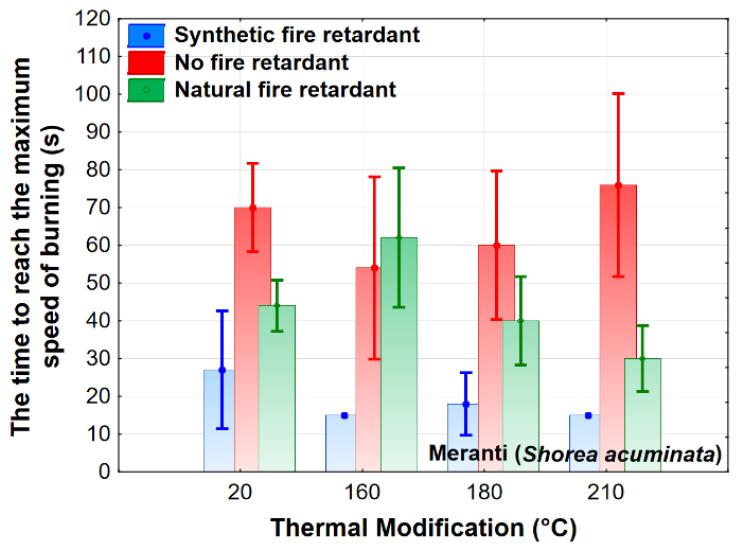
The effect of thermal modification and fire retardant on the time to reach the maximum burning rate.

In our research, between the thermal modification and the time to reach the maximum burning rate, the synthetic retardant had a very statistically significant effect on the maximum burn rate, up to two times lower values compared to treatment a with natural retardant. The observed characteristics for both retardants are recorded in (Figure 5), where they are shown on the blue and green curves we can see a similar trend for this characteristic at temperatures of 160 °C and 180 °C.

The results evaluating the effect of the observed factors on combustion characteristics were also evaluated using Duncan’s tests. The test results fully confirm the results of Figure 1, Figure 2, Figure 3, Figure 4 and Figure 5. Because of the large-scale work, we do not report these results.

During thermal treatment, the content of extractives increases (Table 3). Some extractives disappear or degrade at higher temperatures; however, new components are formed due to the degradation of main wood components, especially hemicelluloses. The rise in the content of extractives is due to the degradation of lignin and polysaccharides [46]. The increase in lignin content at a temperature of 160 °C is caused by condensation reactions with degradation products of polysaccharides and further cross-linking, thus increasing the apparent lignin content, i.e., pseudo-lignin [48,49]. A similar trend was observed in thermally treated teak and iroko [49]; however, at higher temperatures (180 °C and 210 °C), the lignin content declines, probably due to predominant degradation reactions.

Hemicelluloses are the most affected by higher temperatures, and their content decreases by 80% at a temperature of 210 °C when compared to untreated samples. The sharp drop in hemicelluloses between 180 °C and 210 °C can be attributed to the decomposition of xylan, the most thermally unstable hemicellulose [50]. Xylan is the dominant hemicellulose in meranti wood, and it can be quantitatively converted to xylose by dilute acid hydrolysis [51]. The increase in cellulose content is about 12%, probably because of its crystalline nature [51]. It is worth noting that tropical tree hemicelluloses are more thermally unstable compared to temperate tree hemicelluloses. The decrease in hemicellulose content in oak and spruce wood is 58% and 37%, respectively; on the other hand, the drop in hemicellulose content in teak, meranti, and merbau wood is 67%, 80%, and 90%, respectively [44,45,52]. This phenomenon may be due to the different structure of hemicelluloses in tropical and temperate wood species, e.g., molecular weight, branching, etc.

The results of the Spearman correlation (Table 5) showed a high degree of dependence between the burning properties and the chemical components of the wood. The chemical components of the wood and the temperature of the thermal modification have reached very high values of a degree of dependence of more than 99%. The combustion characteristics had a moderate degree of dependence in interaction with the chemical components of the wood. The dependency ranged from −31% to 28%.

## 4. Conclusions

The effect of synthetic fire retardant proved to be a statistically very significant agent, influencing the combustion process in all monitored characteristics. These findings are comparable with the results of other works.

Weight loss values can be reduced by impregnating the wood matrix with synthetic flame retardant (ammonium phosphate). In contrast, no significant effect on weight loss values was observed related to the reference sample treated with a natural flame retardant (arabinogalactan).

The effect of the temperature modification on the burning rate had a similar course as the weight loss. The thermal modification had a significant effect on the burning rate with all values increased. There was a similar course to the previous characteristic, and there was no statistically significant difference between the temperatures. A significant positive effect on the reduction of the combustion rate at all recorded temperatures for samples treated with synthetic flame retardant compared with non-treated samples was observed again. A statistically significant effect at higher temperatures (180 °C and 210 °C), with a decrease of the burning rate values of the treated wood, was observed.

A significant reduction of burning rate for synthetic retardant treated samples was observed. After the application of the natural retardant, there was a decrease in the values of the maximum burning rate, however not so significant related to those synthetic.

Heat-treated samples treated with synthetic retardant also had a significant statistical effect on the maximum burning rate ratio. After application of the natural retarder or between the temperatures of the heat treatment itself, there was an increased resistance of the synthetic retardant compared to the natural thermal modification at 160 and 210 °C, however, this effect was not statistically proven. The differences between the effects of synthetic and natural flame retardant on the burning properties of heat-treated meranti wood were studied. Due to the heterogeneity of the wood material and better interpretation of the data in further research, we will choose more repetitions for measurement in order not to achieve such a high coefficient of variation.

The relative content of the extractives, lignin, and cellulose after the heat treatment was increased, whereas the values of holocellulose and especially hemicelluloses were decreased significantly. The positive effect of thermal modification on the flammability characteristics of meranti wood was not confirmed.

Positive effects on all flame characteristics for samples treated with synthetic retardant compared to those treated with the natural one were described. In the case of arabinogalactan application and its maximum potential positive retarding effect, it is necessary to optimize the conditions of the experiments for its purpose.

## Figures and Tables

**Table 1:** Conditions and Parameters of Thermal Modification.

Thermal Modification Parameters			
**Temperatures (°C)**	160	180	210
**Heating (h)**	13.5	13.6	18.7
**Thermal treatment (h)**	3.0	3.0	3.0
**Cooling (h)**	9.6	11.1	11.2
**Total modification time (h)**	26.1	27.7	32.9

**Table 2:** The density of Shorea Samples.

Temperature	Thermal Modification Temperature			
	Unmodifield	160 °C	180 °C	210 °C
**Density before TM (kg·m^−3^)**	626 (9.3)	662 (11.4)	634 (10.8)	626 (8.5)
**Density after TM (kg·m^−3^)**		625 (9.2)	608 (10.4)	650 (7.2)

Numbers in parentheses represent coefficients of variation (CV) in %.

**Table 3:** Chemical analyses of the main wood components of meranti (%) and the main results of a way ANOVA analysis evaluating the effect of the thermal modification temperature on the values of interest.

**Temperature** **(°C)**	**Extractives** **(%)**	**Fisher’s F-Test** **Level of Significance**	**Lignin** **(%)**	**Fisher’s F-Test** **Level of Significance**	**Cellulose** **(%)**	**Fisher’s F-Test** **Level of Significance**
**Unmodified**	2.51 (2.37)	297.04 ***	32.42 (0.25)	1468 ***	53.04 (0.89)	543.5 ***
**160**	3.22 (1.94)		36.67 (0.26)		51.02 (0.19)	
**180**	3.20 (1.47)		36.29 (0.24)		52.04 (0.31)	
**210**	3.92 (2.32)		35.33 (0.37)		59.06 (0.60)	
**Temperature** **(°C)**	**Holocellulose** **(%)**	**Fisher’s F-Test** **Level of Significance**	**Hemicelluloses** **(%)**	**Fisher’s F-Test** **Level of Significance**		
**Unmodified**	71.53 (0.58)	259.2 ***	18.49 (4.65)	367.925 ***		
**160**	66.51 (1.07)		15.50 (5.14)			
**180**	66.12 (0.23)		14.08 (2.16)			
**210**	62.71 (0.54)		3.65 (6.13)			

The data represent the mean percentages of oven-dry weight (odw), numbers in parentheses represent coefficients of variation (CV) in %, *n* = 4, NS—not significant, ***—significant, *P* < 0.05.

**Table 4:** Basic Statistical Characteristics Evaluation of the Effect of Thermal Treatment and Retardants on the Values of the Monitored Characteristics.

Influence	Sum of Squares	Degrees of Freedom	Variance	Fisher’s F-Test	Significance Level *P*
WL.—600 s (%)					
**Intercept**	1495.404	1	1495.404	1430.428	***
**Retardant**	374.391	2	187.196	179.062	***
**TM**	14.324	3	4.775	4.567	***
**Retardant × TM**	17.222	6	2.870	2.746	***
**Error**	50.180	48	1.045		
The model corresponds to approximately 89.0% of the total sum of squares.					
BR.—600 s (%·s^−1^ × 10^−5^)					
**Intercept**	17.66190	1	17.66190	174.4060	***
**Retardant**	1.38970	2	0.69485	6.8615	***
**TM**	0.13538	3	0.04513	0.4456	NS
**R × TM**	0.79910	6	0.13318	1.3151	NS
**Error**	4.86091	48	0.10127		
The model corresponds to approximately 32.3% of the total sum of squares.					
MBR. (%·s^−1^)					
**Intercept**	531.7951	1	531.7951	1244.976	***
**Retardant**	50.8410	2	25.4205	59.511	***
**TM**	2.3821	3	0.7940	1.859	NS
**Retardant × TM**	4.6048	6	0.7675	1.797	NS
**Error**	20.5033	48	0.4272		
The model corresponds to approximately 73.8% of the total sum of squares.					
RMBR. (%)					
**Intercept**	0.456095	1	0.456095	558.1834	***
**Retardant**	0.036244	2	0.018122	22.1784	***
**TM**	0.019321	3	0.006440	7.8817	***
**Retardant × TM**	0.026381	6	0.004397	5.3810	***
**Error**	0.039221	48	0.000817		
The model corresponds to approximately 67.6% of the total sum of squares.					
TRMBR.					
**Intercept**	108,800.4	1	108,800.4	464.2151	***
**Retardant**	21,450.8	2	10,725.4	45.7618	***
**TM**	544.6	3	181.5	0.7745	NS
**Retardant × TM**	4079.2	6	679.9	2.9007	***
**Error**	11,250.0	48	234.4		
The model corresponds to approximately 69.9% of the total sum of squares.					

NS—not significant, ***—significant, *P* < 0.05, TM.—Thermal modification, WL.—Weight loss, BR.—Burn rate, MBR.—Maximum burn rate, RMBR.—Ratio of the maximum burn rate, TRMBR.—Time to reach the maximum burn rate.

**Table 5:** Spearman’s correlation between burning characteristics and chemical wood components.

Variable	FR.	TM.	WL.	BR.	MBR.	RMBR.	TRMBR.	Ex.	Li.	Hol.	Cell.
FR											
TM.	0										
WL.	90	−4									
BR.	14	10	23								
MBR.	81	17	86	24							
RMBR.	−5	32	3	36	19						
TRMBR.	48	−14	43	−17	40	−79					
Ex.	0	84	−3	3	18	28	−9				
Li.	0	19	13	6	2	11	−8	34			
Hol.	0	−97	4	−11	−17	−32	14	−84	−19		
Cell.	0	39	−16	−1	8	9	−1	28	−77	−41	
Hemicell.	0	−97	4	−11	−17	−31	13	−83	−19	99	−42

Legend: FR.—Retardant, TM.—Thermal modification, WL.—Weight loss, BR.—Burning rate, MBR.—Maximum burning rate, RMBR.—The ratio of the maximum burning rate, TRMBR.—Time to reach the maximum burning rate, Ex.—Extractives, Li.—Lignin, Hol.—Holocellulose, Cell.—Cellulose, Hemicell.—Hemicelluloses.

## Data Availability

The data presented in this study are available on request from the corresponding author.
